# Excitation-Dependent pKa Extends the Sensing Range of Fluorescence Lifetime pH Sensors

**DOI:** 10.3390/s24237531

**Published:** 2024-11-26

**Authors:** Emily P. Haynes, Mary Canzano, Mathew Tantama

**Affiliations:** 1 Department of Chemistry, Purdue University, 560 Oval Drive, West Lafayette, IN 47907, USA; 2 Department of Chemistry, Wellesley College, 106 Central Street, Wellesley, MA 02481, USA; 3 Biochemistry Program, Wellesley College, 106 Central Street, Wellesley, MA 02481, USA

**Keywords:** pH, fluorescence lifetime, pKa

## Abstract

Biological activity is strongly dependent on pH, which fluctuates within a variety of neutral, alkaline, and acidic local environments. The heterogeneity of tissue and subcellular pH has driven the development of sensors with different pKa values, and a huge assortment of fluorescent sensors have been created to measure and visualize pH in living cells and tissues. In particular, sensors that report based on fluorescence lifetime are advantageous for quantitation. Here, we apply a theoretical framework to derive how the apparent pKa of lifetime-based pH sensors depends on fluorescence excitation wavelength. We demonstrate that theory predicts the behavior of two different fluorescent protein-based pH sensors in solution as proofs-of-concept. Furthermore, we show that this behavior has great practical value in living cells because it extends the sensing range of a single sensor by simply choosing appropriate detection parameters to match the physiological pH range of interest. More broadly, our results show that the versatility of a single lifetime-based sensor has been significantly underappreciated, and our approach provides a means to use a single sensor across a range of pH environments.

## 1. Introduction

The pH of an aqueous environment profoundly affects biological activity by determining the charge states of molecules, physical interactions, and chemical reactivity. Furthermore, pH varies substantially across tissues and subcellular compartments [1], and dysregulated pH is implicated in cancer [2], neurological disorders [3], and immunity [4]. This central importance of pH in biochemistry has resulted in a large catalog of measurement techniques, and in particular fluorescent sensors continue to be developed to meet the challenge of pH quantification and visualization in living systems that include whole model organisms [5,6,7,8].

Despite the impressive array of available tools, often using just one fluorescent sensor alone is insufficient to assess the huge variation of pH in and around a cell using conventional detection protocols. Instead, parallel experiments must be conducted with a set of sensors, each with its own pKa to match a respective compartment [9]. Alternatively, a multiplexed experiment can measure several compartments simultaneously using differently colored fluorescent sensors together, again each with its own characteristic pKa. While these approaches have become standard, they require the loading or expression of multiple sensors, and multiplexing can occupy a large bandwidth of spectral emission space, limiting the use of sensors for other physiological parameters of interest. To this end, a single-emission color pH sensor with a broad pH sensing range would be advantageous.

Currently, there are many single-color sensors that report pH changes with a change in fluorescence intensity [9,10], but these require careful calibration because the absolute intensities depend on sensor expression level. In contrast, single-color sensors that report pH dynamics with changes in fluorescence lifetime respond independently from sensor concentration and are invariant to photobleaching, providing a robust advantage for quantitative comparisons [11,12,13,14,15]. One caveat to lifetime sensors is that the analyte concentration for half-maximal lifetime change *K^app^* is determined not only by the sensor-analyte equilibrium but also the brightness and photon statistics of the sensor itself [16]. This is an important aspect to the quantitative operation and interpretation of lifetime sensors. While this is considered by most as a cautionary note on how to use lifetime sensors properly, earlier work by Szmacinski and Lakowicz showed this can be leveraged to expand the calcium sensing range of Fura-2 [17].

Here, we present a theoretical framework that accounts for photon-counting statistics to predict how the apparent pK_a_ of a fluorescence lifetime pH sensor depends on the excitation wavelength used for illumination. Rather than a peculiarity or nuisance, we show this offers an important practical advantage in extending the pH sensing range of a single sensor.

## 2. Materials and Methods

### 2.1. Protein Expression and Purification

Polyhistidine-tagged mCherryTYG and pHRed fluorescent proteins were expressed in BL21(DE3) *E. coli* grown in auto-induction media shaking overnight at 37 °C in baffled flasks followed by an additional 3–4 days shaking at room temperature to allow for maximal maturation of their chromophores. Cell pellets were collected by centrifugation and lysed by sonication. Protein was purified by nickel-affinity chromatography, followed by dialysis and concentration. Protein stocks were snap-frozen in liquid nitrogen and stored at −80 °C until use.

### 2.2. Time-Resolved Fluorescence Spectroscopy

Fluorescence lifetimes were measured by time-correlated single photon counting on an Edinburgh Instruments FS5-TCSPC+ spectrophotometer. Samples were excited using an NKT-Fianium WhiteLaseMicro supercontinuum pulsed laser with a 20 MHz repetition rate and an Edinburgh Instruments excitation wavelength selector. Photon count rates were maintained at 250,000 counts per second or less in order to avoid photon pileups. Samples were continuously stirred at room temperature.

Fluorescence decay single-photon count histograms were collected using 1 µM purified protein (mCherryTYG or pHRed) in solutions buffered from pH 5.5 to 9.0 in duplicates. Instrument response functions (IRFs) were measured for each excitation wavelength using a Ludox suspension (Figure S1). Edinburgh Instruments Fluoracle and FAST 3.4.2 software was used for reconvolution fitting in order to account for the wavelength-dependent IRFs. Single-photon decays were iteratively fitted to one to four exponential decay components until the chi-square decreased by less than 5%, and chi-square values were on average less than 1.5. Mean lifetimes reported for purified proteins are the weighted average of the fitted decay components. Lifetime versus pH data at each wavelength were fitted to a Boltzmann function in OriginPro to determine mean pK_a_ values and standard errors.

Time-course measurements of the fluorescence decays of live cells were collected from DH5a *E. coli* expressing pHRed. As previously described, leaky expression of the T7 promoter is sufficient for expression in DH5a cells without induction, and this method achieved reproducible expression levels [18,19]. Cells were grown in Luria Broth shaking overnight at 37 °C and then shaken for an additional 3–4 days at room temperature before use. Each replicate represents an independent culture. Cells were washed and re-suspended in glucose-free M9 media buffered with MES adjusted to pH 6 with KOH. At each time point, fluorescence lifetimes were collected by sequentially exciting the sample at 440 nm and 575 nm. After five minutes of baseline measurements, 10 mM glucose was added to the suspension culture to initiate a fuel-dependent alkalinization of the cytosol, and at 10 min, 1 mM KCN was added to block respiration and the fuel-dependent alkalinization [18]. For live-cell measurements, the tail mean lifetime was measured. Although the tail mean lifetime is convoluted with the IRF, the IRF did not vary significantly across any conditions, and the tail mean lifetime correlates with the mean fitted lifetime [18,19].

## 3. Results and Discussion

### 3.1. Theory

We apply the framework developed by Yellen and co-workers [16] to fluorescence lifetime pH sensors. Fluorescent protein-based pH sensors exhibit a change in fluorescence spectra and fluorescence lifetime that are dependent on the protonation state of the fluorescent protein chromophore (*Cro*) [12,15,18,19]. The chromophore contains a tyrosine-derived hydroxyl group that can change the protonation state within physiological pH ranges, and thus we assume a single-site protonation model [20,21]. Let *CroH* and *Cro^−^* denote the protonated and deprotonated chromophore states. Typically, the deprotonated anionic *Cro^−^* state is associated with a brighter and longer-lifetime state with a longer wavelength absorbance peak, and the protonated neutral *CroH* state is associated with a dimmer and shorter lifetime state with a shorter wavelength absorbance peak [18,20,21,22]. Let *F_H_* and *F_−_* denote the fluorescence intensity for the protonated and deprotonated states, respectively. Note that the fluorescence intensity, or number of emitted photons, is proportional to the product of the fluorescence quantum yield and extinction coefficient, ϕ_f_·ε_λ_, and varies with excitation wavelength λ_ex_. Let *τ_H_* and *τ*_−_ denote the fluorescence lifetimes of the protonated and deprotonated states. We can then write the following fractional occupancy of the protonated state, denoted as *y*:

(1) $$y=\frac{\left[CroH\right]}{\left[CroH\right]+\left[{Cro}^{-}\right]}=\frac{1}{1+\frac{\left[{Cro}^{-}\right]}{\left[CroH\right]}}$$

We can also write the Henderson–Hasselbalch equation as follows:

(2) $$pH={pK}_{a}+{log}_{10}\left(\frac{\left[{Cro}^{-}\right]}{\left[CroH\right]}\right)$$

Combining Equations (1) and (2), we can re-write the fractional occupancy in the equation with familiar form as follows:

(3) $$y=\frac{1}{1+10^{\left(pH-{pK}_{a}\right)}}$$

Now, at a given excitation wavelength, we can measure the pH-dependence of the fluorescence lifetime and determine “*x*”, the pH at which there is a half-maximal change in lifetime, as shown the following Equation (4):

(4) $$\tau_{x}=\frac{\tau_{H}+\tau_{-}}{2}$$

By Kasha’s Rule, the fluorescence quantum yield and intrinsic fluorescence lifetime would not be expected to vary significantly with excitation wavelength over a narrow energy band in the visible range that accesses the same final S_0_-S_1_ electronic transitions. However, the observed fluorescence lifetime from a solution measurement can vary significantly with excitation wavelength because an ensemble measurement gives the population average of the lifetimes of the two ionization states weighted by the number of photons emitted from each state (i.e., the fluorescence intensities). This is parallel to the excitation wavelength-dependent apparent affinities for the Peredox NADH/NAD^+^ sensor and Fura-2 calcium sensor that were observed by Mongeon and Szmacinski, respectively [16,17]. Thus, we can write Equation (5):

(5) $$\tau_{x}=\frac{F_{H}\cdot y\cdot \tau_{H}+F_{-}\cdot (1-y)\cdot \tau_{-}}{F_{H}\cdot y+F_{-}\cdot (1-y)}$$

Setting Equations (4) and (5) equal to one another and solving for the fractional occupancy *y*, we find the following:

(6) $$y=\frac{F_{-}}{F_{H}+F_{-}}$$

Finally, combining Equations (3) and (6), we can solve for *x*, the pH at which the half-maximal change in lifetime is measured, which is the apparent *pK_a_^app^*:

$$y=\frac{1}{1+10^{\left(x-{pK}_{a}\right)}}=\frac{F_{-}}{F_{H}+F_{-}}$$

(7) $$x={pK}_{a}^{app}={pK}_{a}+{log}_{10}\left(\frac{F_{H}}{F_{-}}\right)$$

Thus, the apparent *pK_a_^app^* measured by transient fluorescence lifetime spectroscopy is shifted from the true *pK_a_* by an additive factor dependent on the relative brightness of the protonated and deprotonated states when excited at a given wavelength. This brightness ratio can be determined by steady-state spectroscopic measurements of fluorescence excitation spectra in the protonated and deprotonated states.

### 3.2. Experimental Proof-of-Concept for Intensiometric Sensors

**Figure 1:**
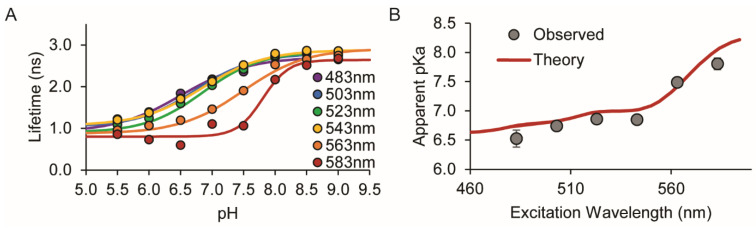
Excitation wavelength-dependent pKa of the intensiometric fluorescent pH sensor mCherryTYG. (**A**) Lifetime-pH titrations of purified mCherryTYG protein in solution. Fitted lines: 483 nm (purple), 503 nm (blue), 523 nm (green), 543 nm (yellow), 563 nm (orange), 583 nm (red). (**B**) Agreement between the theoretical prediction for mCherryTYG using Equation (7) (red line) and experimental observations (circles) for *pK_a_^app^* versus excitation wavelength. Error bars are standard errors from fitting.

In order to test the theory, we first expressed and purified the mCherryTYG pH sensor for solution studies. The mCherryTYG mutant (M66T) of the mCherry red fluorescent protein is a non-ratiometric pH sensor with a single excitation peak that exhibits a decrease in fluorescence intensity with decreasing pH [23]. We previously demonstrated that mCherryTYG is an excellent lifetime sensor with a 2 ns dynamic range and an apparent *pK_a_^app^* of 6.8 with an excitation peak at approximately 543 nm [18]. Here, we measured lifetime titration curves for pH 5.5 to 9.0 using excitation wavelengths at 20 nm increments from 483 nm to 583 nm (Figure 1 and Figure S2).

The titration curves shift right on the graph as the excitation wavelength increases, indicating that the apparent *pK_a_^app^* increases. Importantly, the experimentally determined *pK_a_^app^* values from the fitted titration curves agree well with the predicted *pK_a_^app^* values from theory (Figure 1). Even taking the experiments on their own, this demonstrates a proof-of-concept that mCherryTYG actually has a much broader sensing range, effectively extending from pH 5.5 to 9.0 by varying excitation wavelength.

Having validated the theoretical framework with these experiments, we considered practical limitations. One challenge with a non-ratiometric pH sensor such as mCherryTYG is that, although off-peak excitation extends the sensing range, there is a tradeoff in brightness. At excitation wavelengths much shorter than the peak wavelength, the sensor is much dimmer, independent of the lifetime. As a result, higher illumination intensity and longer emission collection times are required, which is experimentally undesirable. A remedy to this problem could be the use of a ratiometric sensor that exhibits two excitation peaks and remains bright over a broader range of excitation wavelengths because the theory still applies to ratiometric sensors.

### 3.3. Experimental Proof-of-Concept for Ratiometric Sensors

**Figure 2:**
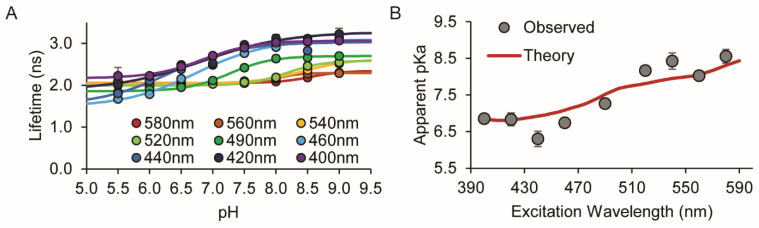
Excitation wavelength-dependent pKa of the ratiometric fluorescent pH sensor pHRed. (**A**) Lifetime-pH titrations of purified pHRed protein. Fitted lines: 400 nm (purple), 420 nm (navy), 440 nm (blue), 460 nm (teal), 490 nm (green), 520 nm (light green), 540 nm (yellow), 560 nm (orange), 580 nm (red). (**B**) Agreement between the theoretical prediction for pHRed using Equation (7) (red line) and experimental observations (circles) for *pK_a_^app^* versus excitation wavelength. Error bars are standard errors from fitting.

To test that the theory can be applied in practice using a ratiometric pH sensor, we used the red fluorescent protein pHRed in solution studies. We previously demonstrated that pHRed has a 0.4 ns dynamic range with an apparent lifetime *pK_a_^app^* of 6.9 using two-photon excitation at 830 nm, and it has one-photon excitation peaks at approximately 440 nm and 575 nm [19]. Here, we measured lifetime titration curves for pH 5.5 to 9.0 using excitation wavelengths at 20 nm increments from 400 nm to 580 nm (Figure 2 and Figure S3).

The individual titration curves are more complex given two strongly observable sensor states in the population average, but despite this, there is still a clear right shift with increasing excitation wavelength, indicating that the apparent *pK_a_^app^* increases. pHRed is interesting in that it has an “inverse” pH dependence in which the anionic chromophore with peak excitation at 575 nm is favored at low pH while the protonated chromophore with peak excitation at 440 nm is favored at high pH. This is accounted for in the theoretical framework by simply swapping the *F_H_* and *F*_−_ terms in Equation (5). With this minor adaptation, we see that the experimentally determined *pK_a_^app^* values from the fitted titration curves generally agree with the predicted *pK_a_^app^* values from theory (Figure 2). However, the differences between experiment and theory may be caused by our instrument, which uses a supercontinuum white light pulsed laser and filter-based wavelength selection. At each center wavelength, there is actually a non-linear mixture of excitation wavelengths. As a result, each experimental lifetime measurement represents a non-linear average of lifetimes, whereas the simplified theory we present assumes perfect single-wavelength excitation. Despite this, and most importantly, the experiment shows there is a clear excitation-dependent *pK_a_^app^* that extends the pH sensing range of pHRed, demonstrating this property is general to both non-ratiometric and ratiometric sensors that exhibit a lifetime change.

### 3.4. Improved Sensing Range in Practice with Live-Cell Spectroscopy

Having validated the theoretical framework and demonstrated experimental proofs-of-concept with solution studies, we considered the practical application in living cells. We chose pHRed as a test case because it has a smaller lifetime dynamic range that more greatly restricts its sensing range when using a single excitation wavelength.

Previously, we demonstrated that fuel-starved *E. coli* respond to glucose feeding with acute intracellular alkalinization correlating with cellular respiration [18]. At baseline, cells in pH 6 media without a carbon source exhibit a cytosolic pH in the 6.5 to 7.0 range. After the addition of glucose, cytosolic pH increases to 8.0 within minutes, and subsequent respiratory blockade with cyanide causes an immediate re-acidification of the cytosol [18]. We used this as our experimental paradigm, in which physiological pH varies from 6.5 to 8.0.

**Figure 3:**
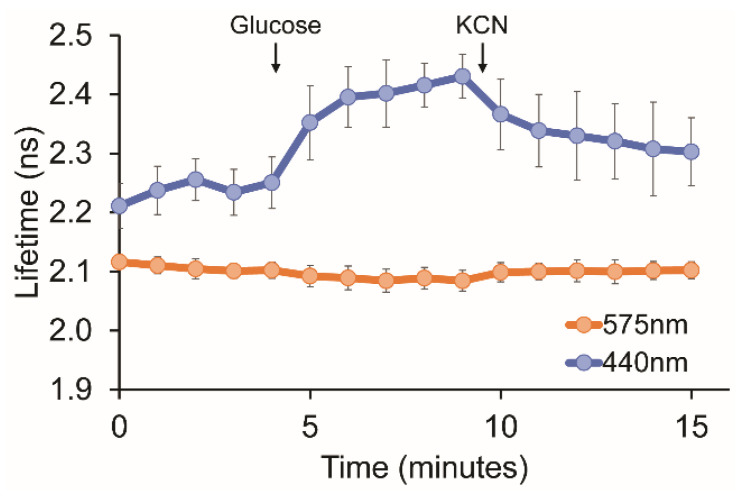
Improved sensitivity of live-cell pH measurements by selection of excitation wavelength. Live *E. coli* expressing pHRed were suspended in media buffered at pH 6.0, lacking any carbon fuel source. After the 4 min time point, glucose was added to fuel cellular respiration, and after the 9 min time point, potassium cyanide was added to block respiration (*n* = 7 from three independent cultures, error bars for 95% confidence interval). Lifetime measurements were made with 440 nm (blue) and 575 nm (orange) excitation.

Suspension cultures of live *E. coli* expressing pHRed were pre-starved and then treated with glucose and cyanide in sequence (Figure 3 and Figure S4). The lifetimes of cell suspensions were continuously measured using alternating 440 nm and 575 nm excitation on the same sample for direct comparison.

Red fluorescent proteins are excited with a 561 nm or 594 nm laser line in a typical confocal microscope found in a core imaging facility. However, with a similar excitation wavelength of 575 nm used here, the apparent *pK_a_^app^* of pHred is near 8.5 and would not be expected to be sensitive to changes in the pH 6.5 to 8.0 range. Indeed, using 575 nm excitation, no change in lifetime was observed for pHRed-expressing cells when either glucose or cyanide was added. In contrast, using 440 nm excitation, the apparent *pK_a_^app^* of pHRed is near 6.5 and would be expected to be much more sensitive to changes in the 6.5–8.0 pH range. As expected, with 440 nm excitation, the lifetimes increased immediately with the addition of glucose due to fuel-dependent respiration, and the lifetimes subsequently decreased with the addition of cyanide that blocks respiration. Thus, the proper choice of excitation wavelength has great practical value in enabling one sensor to be tuned to a desired pH range of interest simply by changing the instrumental parameters.

## 4. Conclusions

We demonstrated that one single pH sensor can exhibit different apparent *pK_a_^app^* values depending on the chosen excitation wavelength using protein solution studies, and we demonstrated that in practice with live cells this property can be exploited to extend the sensing range and greatly improve sensitivity to pH fluctuations more broadly. These results suggest that simple modifications of excitation protocols could reduce the number of pH sensors or parallel experiments required to understand acid–base physiology in real-time. By alleviating the burden of using multiple sensors, the color space can be made available for other lifetime sensors, such as those for ATP and redox status [3,10,13,14,18,19]. Furthermore, this should be a general property of lifetime sensors, not just pH and calcium sensors [17], meaning that our current toolset of sensors may have much broader sensing ranges and therefore applications than previously appreciated.

## Data Availability

Data are available upon request.

## Supplementary Materials

The following supporting information can be downloaded at: https://www.mdpi.com/article/10.3390/s24237531/s1; Figure S1: Instrument response function; Figure S2: mCherryTYG lifetime decays; Figure S3: pHRed lifetime decays; Figure S4: Lifetime decays of pHRed expressed in bacteria during metabolic challenge.
