# Supplementary material for: Excitation-Dependent pKa Extends the Sensing Range of Fluorescence Lifetime pH Sensors
Source: Sensors (Basel). 2024 Nov 26;24(23):7531. doi: 10.3390/s24237531 (PMC11644319; doi:10.3390/s24237531)
Supplement: Supplementary file 1 [file sensors-24-07531-s001.zip › sensors-3285828-supplementary.pdf]

TITLE: Excitation-Dependent pKa Extends the Sensing Range of Fluorescence Lifetime pH Sensors

AUTHORS: Emily P. Haynes
Mary Canzano
Mathew Tantama, mt4@wellesley.edu

SUPPLEMENTARY MATERIALS

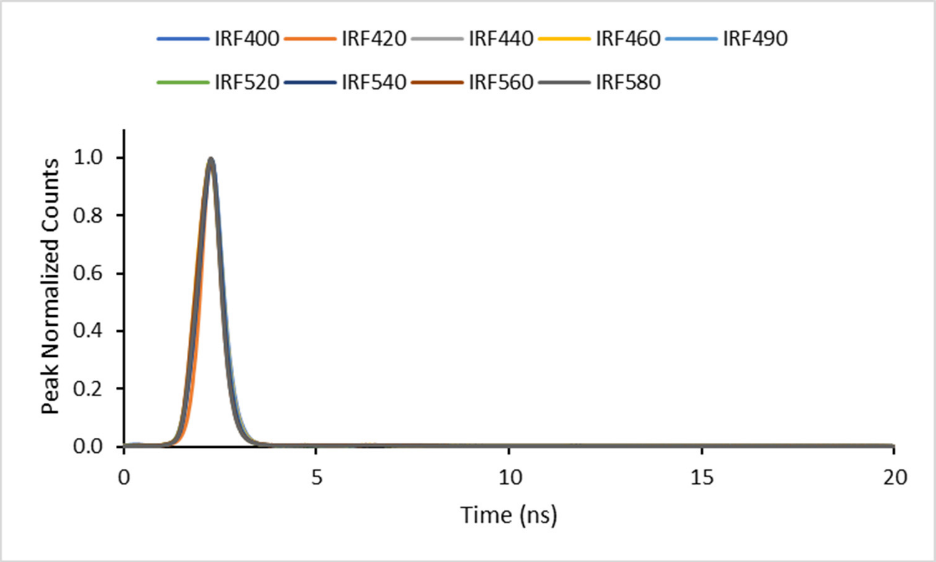

Figure S1. Instrument response functions did not differ significantly across wavelength settings.

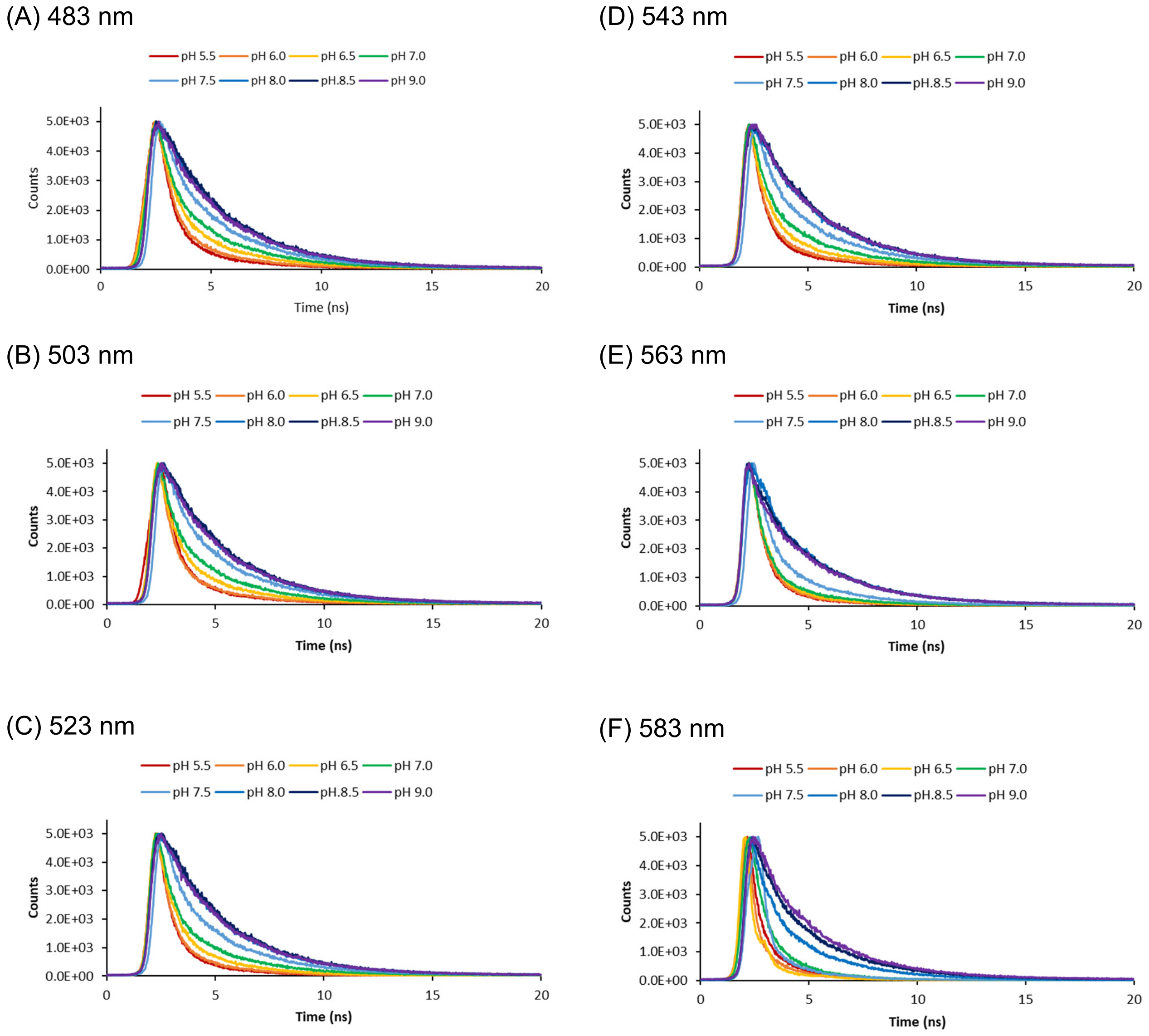

Figure S2. Excitation wavelength and pH dependent fluorescence lifetime decays of mCherryTYG (mCherryM66T) purified protein.

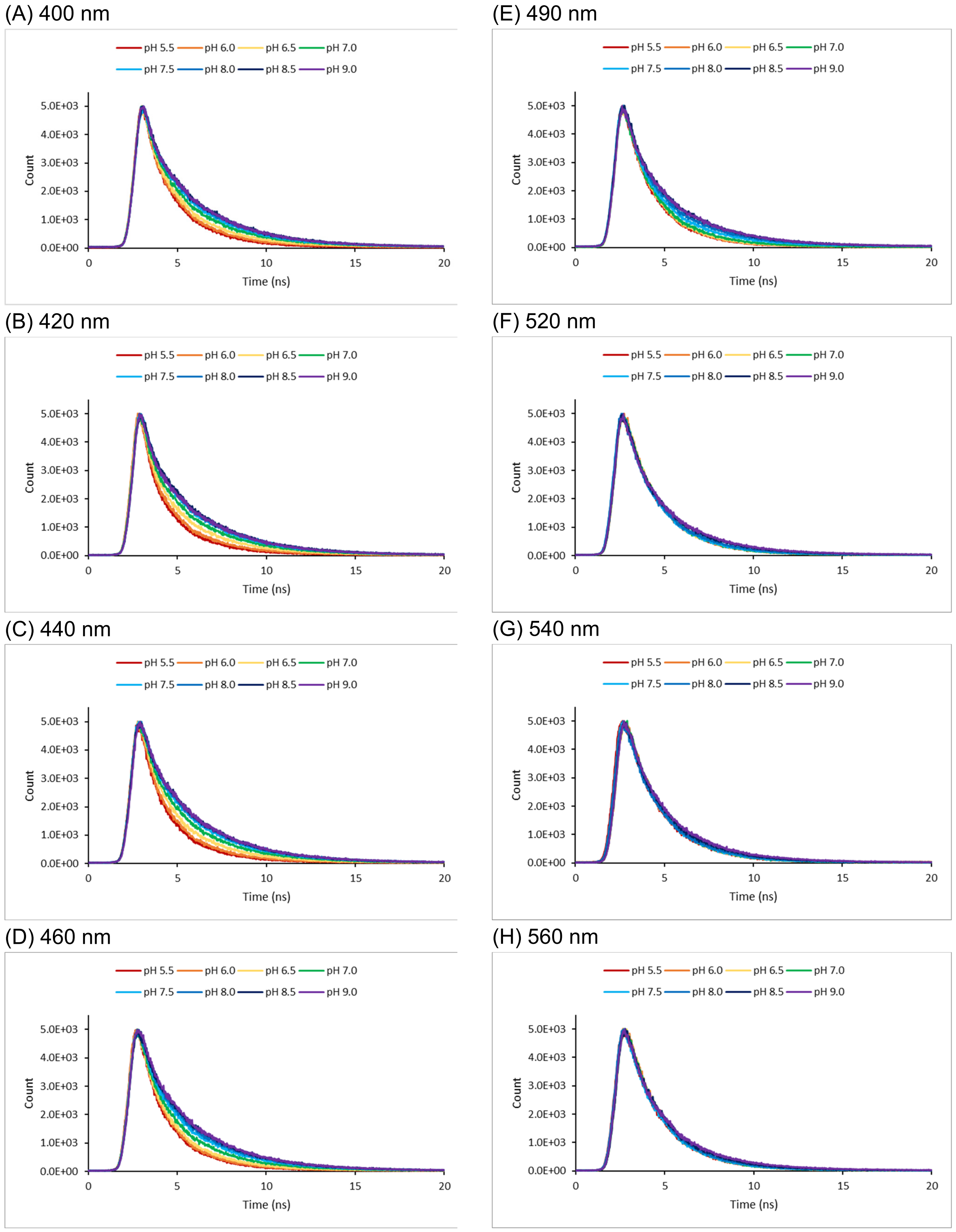

(A) 400 nm
(B) 420 nm
(C) 440 nm
(D) 460 nm
(E) 490 nm
(F) 520 nm
(G) 540 nm
(H) 560 nm
pH 5.5
pH 6.0
pH 6.5
pH 7.0
pH 7.5
pH 8.0
pH 8.5
pH 9.0
Count
Time (ns)
0.0E+00
1.0E+03
2.0E+03
3.0E+03
4.0E+03
5.0E+03
0
5
10
15
20

(I) 580 nm

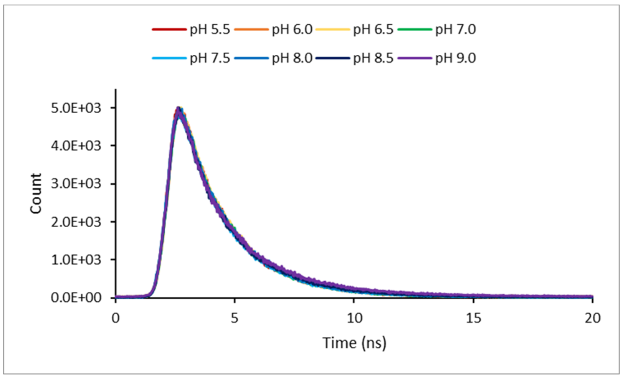

Figure S3. Excitation wavelength and pH dependent fluorescence lifetime decays of pHRed purified protein.

(A) 575 nm (B) 440 nm

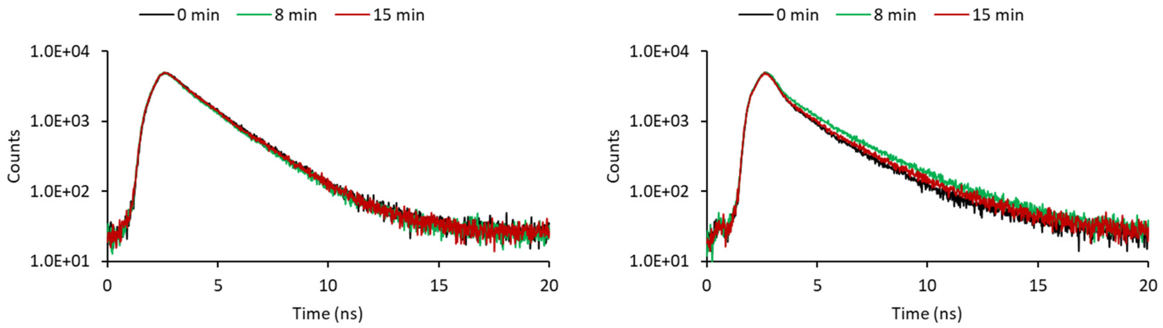

Figure S4. Fluorescence lifetime decays of pHRed expressed in live bacteria during metabolic challenge. *E. coli* were pre-starved at time 0 min. They were then fed glucose, causing cytosolic alkalinization at 8 minutes, after which cyanide was added to block metabolism and cause re-acidification by 15 minutes. (A) Excitation at 575 nm is predicted exhibit a pK_a^{app} ~ 8.5 that is insensitive to these metabolic pH changes. Indeed, the representative fluorescence decays do not show any differences over time. (B) In contrast, excitation at 440 nm is predicted to exhibit a pK_a^{app} ~ 6.5, which is sensitive to these metabolic pH changes. Indeed, the representative lifetime decays show an increase in lifetime with glucose at 8 minutes that is reversed by cyanide at 15 minutes.
